# Small-sized, stable lipid nanoparticle for the efficient delivery of siRNA to human immune cell lines

**DOI:** 10.1038/srep37849

**Published:** 2016-11-28

**Authors:** Takashi Nakamura, Moeka Kuroi, Yuki Fujiwara, Shota Warashina, Yusuke Sato, Hideyoshi Harashima

**Affiliations:** 1 Faculty of Pharmaceutical Sciences, Hokkaido University, Kita-12, Nishi-6, Kita-ku, Sapporo 060-0812, Japan

## Abstract

Gene silencing by small interfering RNA (siRNA) is useful for analyzing the functions of human immune cells. However, the transfection of siRNA to human immune cells is difficult. Here, we used a multifunctional envelope-type nanodevice (MEND) containing YSK12-C4 (YSK12-MEND) to efficiently introduce siRNA to human immune cell lines, Jurkat, THP-1, KG-1 and NK92. The YSK12-MEND was transfected to human immune cell lines at a siRNA dose range of 1–30 nM, resulting that maximum gene silencing efficiencies at the mRNA level in Jurkat, THP-1, KG-1 and NK92 were 96%, 96%, 91% and 75%, respectively. The corresponding values for Lipofectamine RNAiMAX (RNAiMAX) were 37%, 56%, 43% and 19%, respectively. The process associated with cellular uptake played a role in effective gene silencing effect of the YSK12-MEND. The small size and high non-aggregability of the YSK12-MEND were advantageous for the cellular internalization of siRNA to immune cell lines. In the case of RNAiMAX, a drastic increase in particles size was observed in the medium used, which inhibited cellular uptake. The YSK12-MEND reported in herein appears to be appropriate for delivering siRNA to human immune cells, and the small particle size and non-aggregability are essential properties.

Our immune system plays an important role in defending against pathogens. In addition, the immune system is also deeply involved in the maintenance of homeostasis^1^ and a breakdown in the immune system can have severe consequences, leading to the development of autoimmune diseases^2^, cancer^3^, cardiovascular diseases^4^, type 2 diabetes^5^ and obesity^6^. Few fields have had a broader or stronger impact on the analysis of pathogenesis, across all areas of medicine, than immunology^7^. Thus, an analysis of functions of immune cells is quite important for overcoming the above disorders. The majority of immunologists use mice as an experimental tool and the study of their immune responses has yielded tremendous insights. However, as 65 million years of evolution might suggest, there are significant differences between mice and humans^8^. We run the risk of overlooking aspects of human immunology that are not observed in mice. Therefore, studies directed at human tissue (blood, cell, tissue etc) are indispensable for understanding human immunology associated with disorders and for the rational and efficient translation of such findings to the clinic. In particular, *in vitro* analyses using human immune cells can be major goal. In this situation, RNA interference (RNAi) is also a powerful tool for the *in vitro* analysis of gene function.

Small interfering RNA (siRNA) technology has become a powerful research tool^9^. Analyses of gene functions have been carried out using siRNA technology, especially *in vitro*. To induce siRNA-mediated gene silencing, the siRNA must reach the cytoplasm of the target cell where it forms an active complex with an RNAi induced silencing complex. However, the physicochemical properties of siRNA, such as its high molecular weight, an anionic charge and hydrophilicity, largely prevent it from passing through the plasma membrane into the cytoplasm. Therefore, delivery systems are required to effectively deliver siRNA to target cells^10^. In the case of *in vitro* experiments, several delivery systems are available for efficiently delivering siRNA, some of which are commercially available (Lipofectamine^®^ RNAiMAX (RNAiMAX), X-tremeGENE, ViaFect^TM^, etc). RNAiMAX is one of the more popular siRNA transfection reagents for *in vitro* gene silencing. However, these delivery systems are not able to induce efficient gene silencing in all cells and gene silencing efficiency largely differs depending on cell type. In particular, delivering siRNA to immune cells, for example T cells, B cells, natural killer (NK) cells, dendritic cells (DC), macrophages and monocytes, is quite difficult, given the currently available technology. Although a non-viral delivery system, which is easily handled, would be desirable, there is only a few report about effective siRNA delivery to human immune cells by non-viral vectors^11^,^12^,^13^.

In a previous study, we developed a multifunctional envelope-type nanodevice (MEND) for use as a non-viral delivery system^14^ and reported that a MEND containing YSK12-C4 lipid (YSK12-MEND) can be used to deliver siRNA to mouse DC^15^. YSK12-C4 is an ionizable-cationic lipid containing unsaturated carbon chains, which facilitate efficient endosomal escape. The use of the YSK12-MEND resulted in a gene silencing efficiency in excess of 90%, with a median effective dose (ED_50_) of 1.5 nM in mouse DC^15^. The gene silencing ability of the YSK12-MEND was much higher than that of RNAiMAX (ED_50_ was 25 nM). In addition, the silencing of suppressor of cytokine signaling 1, an immune suppressive molecule, by the YSK12-MEND drastically enhanced cytokine production in mouse DC, resulting in a significant suppression of tumor growth when it was applied to DC-based therapy against a mouse lymphoma^15^. Therefore, we hypothesized that the YSK12-MEND would be able to induce efficient gene silencing in human immune cells.

The findings of this study confirm that the YSK12-MEND can be used for the transduction of siRNA to human immune cell lines (Jurkat: human T cells, THP-1: human monocytes, KG-1: human macrophages and NK92: human NK cells). The gene silencing efficiency of the YSK12-MEND was substantially higher than that of RNAiMAX and the YSK12-MEND achieved a gene knockdown in excess of 80% in Jurkat, THP-1 and KG-1 cells, without any toxicity. Moreover, the effective gene silencing by YSK12-MEND was due to its high and homogenous cellular uptake. These findings clearly indicate that the YSK12-MEND represents a promising system for delivering siRNA to human immune cell lines, and provides helpful insights for developing delivery systems for siRNA transduction to human immune cells.

## Results

### The gene silencing in human immune cell lines by YSK12-MEND

**Figure 1:**
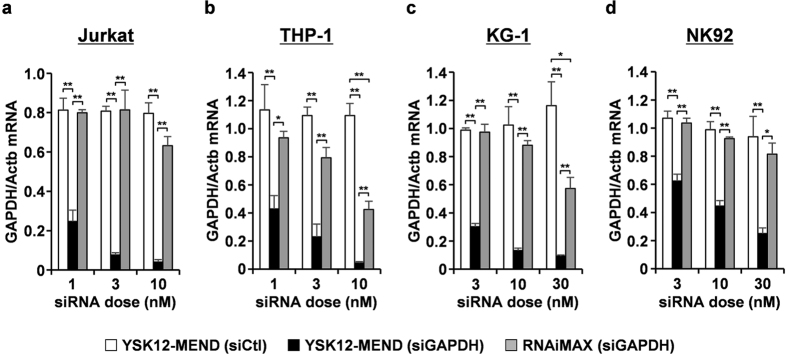
Gene silencing by YSK12-MEND and RNAiMAX in human immune cell lines. Cells were transfected with YSK12-MEND (siCtl), YSK12-MEND (siGAPDH) or RNAiMAX (siGAPDH) at siRNA doses of 1–30 nM. After 24 h, the mRNA levels were measured. (**a**) Jurkat. (**b**) THP-1. (**c**) KG-1. (**d**) NK92. Data are the mean + SEM (n = 3-4, **P < 0.01, *P < 0.05).

The YSK12-MEND used in this study was composed of YSK12-C4, cholesterol, 1,2-dimyristoyl-sn-glycerol methoxyethyleneglycol 2000 ether (PEG-DMG) (85/15/1 mol ratio) and siRNA^15^. Although YSK12-C4 is an ionizable-cationic lipid, the pKa of the YSK12-MEND is 8.0. The YSK12-MEND had a weak cationic charge at a neutral pH. The target siRNA gene was glyceraldehyde-3-phosphate dehydrogenase (GAPDH). We prepared the YSK12-MEND loaded with anti-human GAPDH siRNA (siGAPDH) or control siRNA (siCtl) and investigated the efficiency of gene silencing in Jurkat, THP-1, KG-1 and NK92 cells. RNAiMAX was used as a control, because it represents the most popular and strongest siRNA transfection reagent among the commercially available reagents. The YSK12-MEND induced the significant gene silencing of GAPDH at siRNA doses of 1–10 nM in Jurkat cells and the efficiency was in excess of 90% at siRNA doses of 3 and 10 nM (Fig. 1a). On the other hand, RNAiMAX showed no gene silencing (Fig. 1a). In the case of THP-1 cells, the YSK12-MEND showed the significant gene silencing at siRNA doses of 1–10 nM and the efficiency was in excess of 90% at a siRNA dose of 10 nM (Fig. 1b). RNAiMAX also showed significant gene silencing at a siRNA dose of 10 nM, whereas the efficiency was less than 60% (Fig. 1b). In the case of KG-1 cells, the YSK12-MEND showed significant gene silencing at siRNA doses of 3–30 nM and the efficiency was in excess of 80% at siRNA doses of 10 and 30 nM (Fig. 1c). Although RNAiMAX showed significant gene silencing at a siRNA dose of 30 nM, the efficiency was less than 50% (Fig. 1c). Gene silencing by the YSK12-MEND was observed at siRNA doses of 3–30 nM in NK 92 cells, whereas the efficiencies appeared to be weak compared with Jurkat, THP-1 and KG-1 cells (Fig. 1d). The maximum knockdown efficiency by the YSK12-MEND was 75%. RNAiMAX failed to show significant gene knockdown in NK92 cells (Fig. 1d). Moreover, gene silencing by the YSK12-MEND in Jurkat, THP-1, KG-1 and NK92 cells was observed at the protein level (Fig. S1). Consequently, the YSK12-MEND can be a useful siRNA carrier that is capable of achieving a high degree of gene silencing in Jurkat, THP-1, KG-1 and NK92 cells.

### Evaluation of cytotoxicity by YSK12-MEND in human immune cell lines

**Figure 2:**
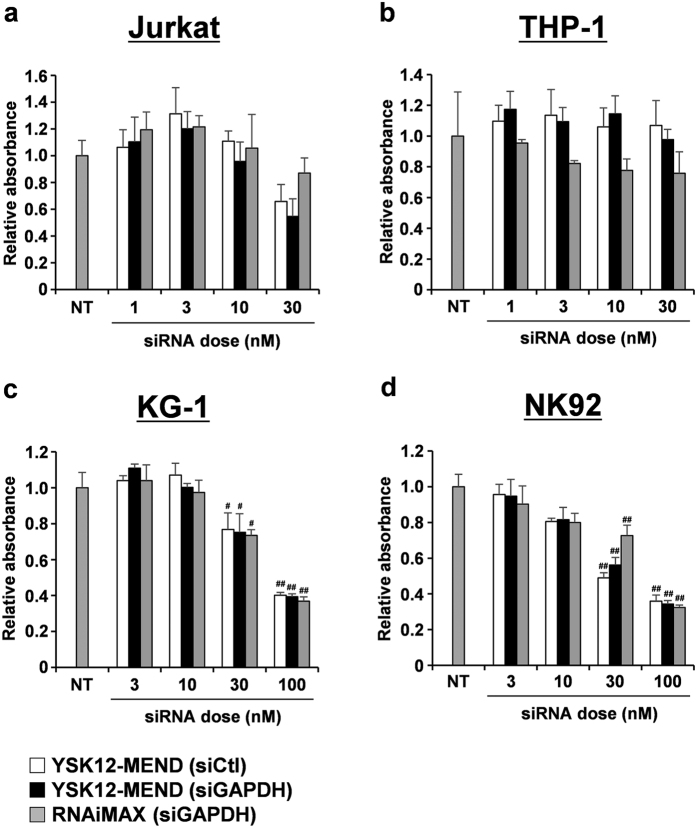
Cytotoxicity by YSK12-MEND and RNAiMAX in human immune cell lines. Cells were transfected with YSK12-MEND (siCtl), YSK12-MEND (siGAPDH) or RNAiMAX (siGAPDH) at siRNA doses of 1–100 nM. After 24 h, the cytotoxicities were analyzed by WST-1 assay. (**a**) Jurkat. (**b**) THP-1. (**c**) KG-1. (**d**) NK92. Data are the mean + SEM (n = 3, **P < 0.01, *P < 0.05 vs no treatment (NT)).

We next examined the cytotoxicity in Jurkat, THP-1, KG-1 and NK92 cells, when the YSK12-MEND was transfected in a siRNA dose range of 1–100 nM. Cytotoxicity was evaluated by mean of a WST-1 assay 24 h after the transfection of the YSK12-MEND. As a result, the transfection of the YSK12-MEND or RNAiMAX to Jurkat cells showed no statistically significant cytotoxicity compared with non-treated (NT) cells, whereas the YSK12-MEND and RNAiMAX appeared to induce a slight cytotoxicity at a siRNA dose of 30 nM (Fig. 2a). In the case of THP-1 cells, the transfection of the YSK12-MEND or RNAiMAX showed no statistically significant cytotoxicity compared with NT cells, whereas the RNAiMAX appeared to induce a slight cytotoxicity at siRNA doses of 10 and 30 nM (Fig. 2b). On the other hand, a statistically significant cytotoxicity was found, compared with NT cells at siRNA doses of 30 and 100 nM in KG-1 and NK92 cells (Fig. 2c and d). These results confirm that the YSK12-MEND is not cytotoxic at the siRNA doses required to achieve sufficient gene silencing in excess of 80% in Jurkat, THP-1 and KG-1 cells. On the other hand, the results for NK92 cells indicate that the YSK12-MEND induces only 55% gene silencing at the siRNA dose that is not cytotoxic.

### Effect of cellular uptake efficiency on gene silencing by YSK12-MEND

**Figure 3:**
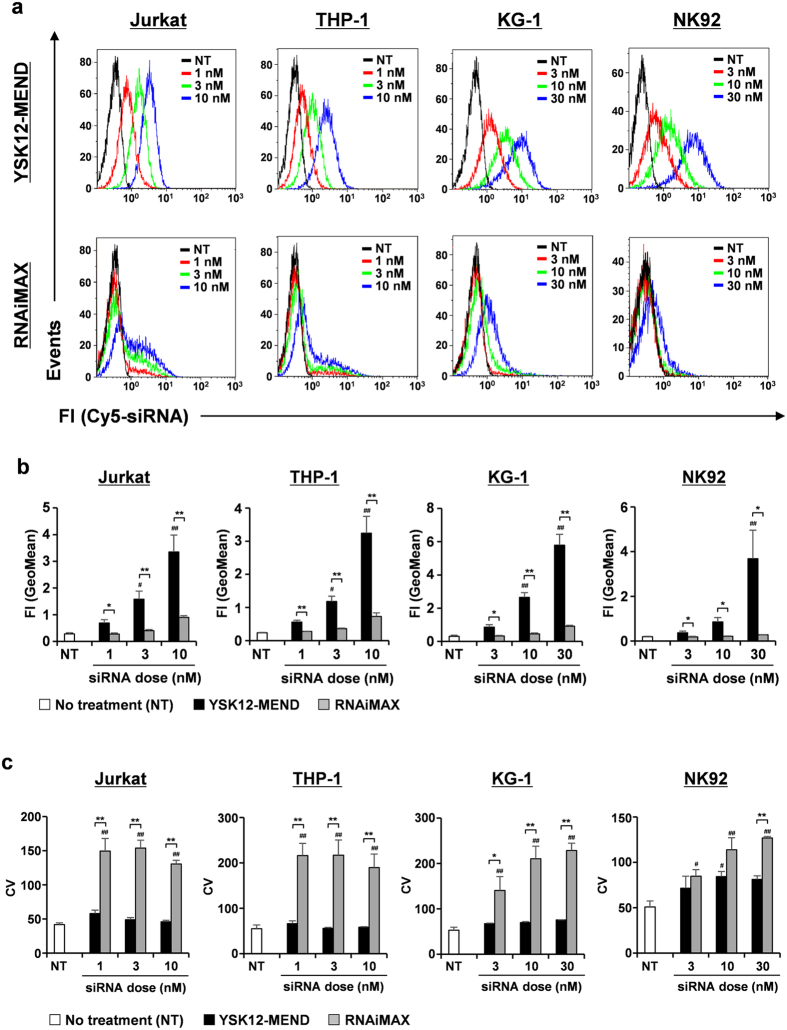
Cellular uptake of the YSK12-MEND and RNAiMAX by human immune cell lines. Cells were transfected with YSK12-MEND (Cy5-siRNA) or RNAiMAX (Cy5-siRNA) at siRNA doses of 1-30 nM. After 2 h, the cells were collected and were analyzed by flow cytometry. (**a**) Typical histograms. (**b**) Average data of GeoMean of FI. (**c**) Average data of CV. Data are the mean + SEM (n = 3, **P < 0.01, *P < 0.05; ^##^P < 0.01, ^#^P < 0.05 vs no treatment (NT)).

The process of the cellular uptake of siRNA greatly influences the gene silencing efficiency. To clarify the factors responsible for the efficient gene silencing by the YSK12-MEND in human cell lines, we investigated the uptake of siRNA by the YSK12-MEND in Jurkat, THP-1, KG-1 and NK92 cells. The cellular uptake was evaluated by flow cytometry 2 h after the transfection of Cy5-labeled siRNA contained by the YSK12-MEND or RNAiMAX. Figure 3a shows typical histograms for Jurkat, THP-1, KG-1 and NK92 cells. These histograms show that the YSK12-MEND was efficiently internalized by each human cell line and the degree of internalization was highly homogenous, compared with RNAiMAX (Fig. 3a). Based on these histograms, we determined the amount of siRNA in the cells, the percentage siRNA contained by the cells and the homogeneity of cellular uptake by using the geometric mean (GeoMean) of the fluorescent intensity (FI), the %gated and the coefficient variation (CV), respectively. The average data are shown in Fig. 3b, S2 and 3c. The amounts of siRNA in cells transfected with YSK12-MEND were significantly higher than the corresponding values for RNAiMAX at all siRNA doses in all cell lines (Fig. 3b). The values of GeoMean in the case of the YSK12-MEND were significantly-high compared with the NT group as follows: 3 and 10 nM siRNA doses in Jurkat and THP-1 cells; 10 and 30 nM siRNA doses in KG-1 cells; 30 nM siRNA dose in NK92 cells (Fig. 3b). This result clearly indicates that the YSK12-MEND efficiently delivers siRNA to human cell lines.

We set a single linear region as shown in Fig. S2a. The single linear regions were set under conditions that the value of %gated in the histogram of NT cells was equal to or less than 1%. As a result, the %gated values for the YSK12-MEND were significantly higher than those of RNAiMAX in all cell lines (Fig. S2a). In this experimental condition, %gated values for the YSK12-MEND approached 100% in all cell lines (Fig. S2a). This result suggests that the YSK12-MEND is capable of delivering siRNA to all cells in human cell lines.

The homogeneity of cellular uptake is so high that CV value is small, because CV value shows a degree of variation. That is, the homogeneity is high, thus making the CV value similar to the CV value for the NT group. In the cases of Jurkat, THP-1 and KG-1 cells, there was no significant difference between the NT group and the YSK12-MEND treated groups, indicating that the cellular uptake by the YSK12-MEND was highly homogenous (Fig. 3c). On the other hand, there was a significant difference between the NT group and RNAiMAX treated groups, and the CV values for the RNAiMAX treated groups were much higher than those for the YSK12-MEND treated groups (Fig. 3c). This fact indicates that the cellular uptake of RNAiMAX was not completely homogenous. In the case of NK92 cells, there was no significant difference between the NT group and the YSK12-MEND treated groups (3 and 30 nM siRNA), whereas the CV value for the YSK12-MEND treated group at a siRNA dose of 10 nM was significantly higher than that of the NT group (Fig. 3c). On the other hand, there was a significant difference between the NT group and the RNAiMAX treated groups. At a siRNA dose of 30 nM, the CV value of the RNAiMAX treated groups was much higher than that of YSK12-MEND treated groups (Fig. 3c). Thus, the cellular uptake of the YSK12-MEND appeared to be highly homogenous compared with RNAiMAX in NK92 cells.

**Figure 4:**
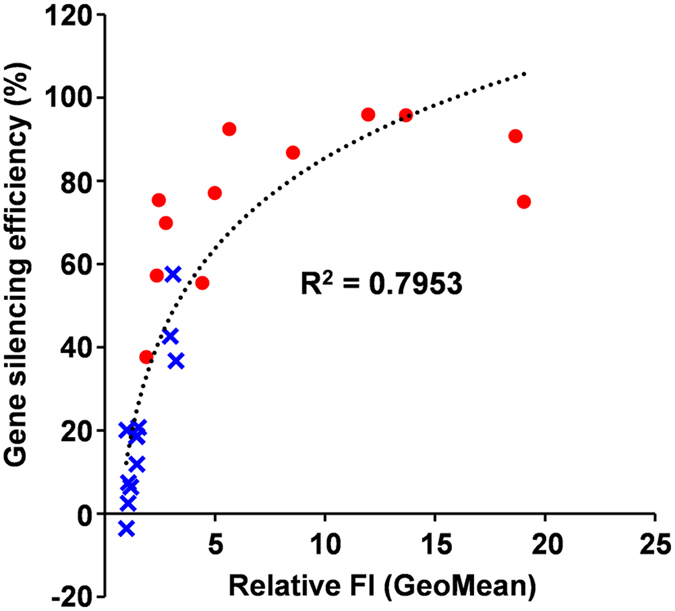
Relationship between gene silencing efficiency and the amount of siRNA (GeoMean of FI). Red circles and blue cross marks show a plot of YSK12-MEND treated cells and the plot of RNAiMAX treated cells, respectively. Dotted line shows the approximated curve.

To investigate the relationship between gene silencing efficiency and the amount of siRNA in the cells (GeoMean of FI), we prepared scatter plots of variables for gene silencing efficiency and the GeoMean of FI. The GeoMean of FI were normalized by the value of NT cells and is shown as relative FI (GeoMean). Figure 4 clearly shows that the gene silencing efficiency is so high that the GeoMean of FI, namely the amount of siRNA in the cells, is high. These results suggest that the efficient gene silencing by the YSK12-MEND is due to efficient cellular uptake.

### The small size and high non-aggregability of YSK12-MEND are advantageous for cellular uptake

**Figure 5:**
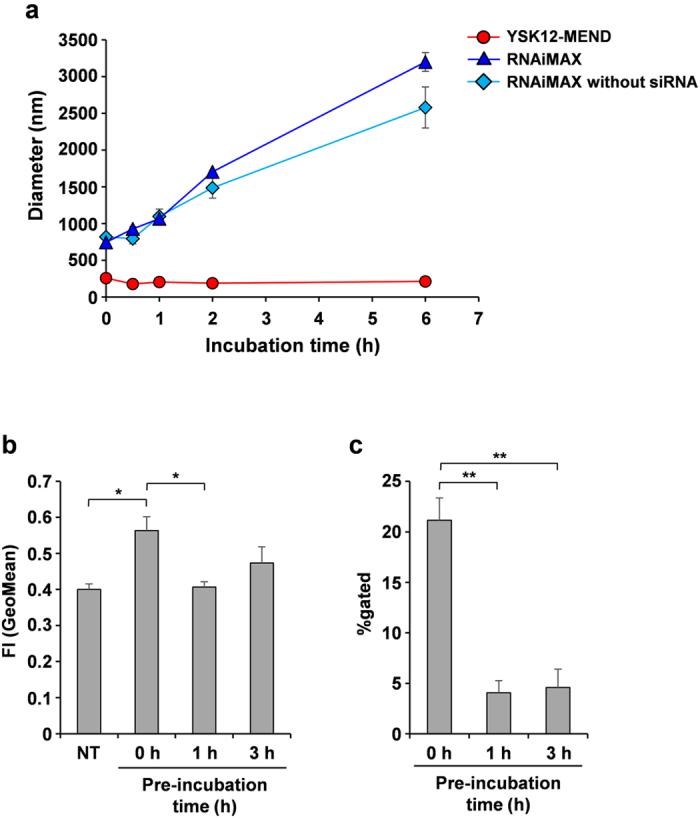
Effect of particle size and non-aggregability on cellular uptake. (**a**)YSK12-MEND and RNAiMAX were incubated in OPTI-MEM and the change in diameter was measured. Data are the mean + SEM (n = 3). (**b**) After the preparation of RNAiMAX (Cy5-siRNA), the RNAiMAX was incubated in OPTI-MEM for 1 h or 3 h. After the incubation, the RNAiMAX was added to Jurkat and the cellular uptake was analyzed by flow cytometry. Data are the mean + SEM (n = 3, **P < 0.01, *P < 0.05).

To identify the cause of the efficient cellular uptake of the YSK12-MEND, We focused on particle size in the transfection media. Changes in the size of YSK12-MEND and RNAiMAX in the OPTI-MEM I medium were investigated. The YSK12-MEND, RNAiMAX and RNAiMAX without siRNA were incubated in the OPTI-MEM I media under the same conditions as were used for siRNA transfection (30 nM siRNA dose) for 0.5, 1, 2 and 6 h, and the sizes of the particles were then measured. The findings showed, the particle size of the YSK12-MEND was not altered in the OPTI-MEM I medium, whereas the particle size of RNAiMAX drastically increased with time (Fig. 5a). In addition, RNAiMAX aggregation occurred in both the presence and absence of siRNA (Fig. 5a). On the other hand, RNAiMAX showed no aggregation and the pattern of the YSK12-MEND was similar to that in HEPES buffer (Fig. S3). The facts indicate that RNAiMAX aggregated in the siRNA transfection process.

We subsequently investigated the effect of the aggregation of RNAiMAX on the cellular uptake of siRNA in Jurkat cells. RNAiMAX was prepared following the manufacturer’s instructions, and was immediately transfected to Jurkat cells (0 h), transfected after a 1 h incubation (1 h) and transfected after a 3 h incubation (3 h). The diameters of RNAiMAX in the case of 0 h, 1 h and 3 h were 1024 ± 143 nm, 2286 ± 357 nm and 3035 ± 873 nm, respectively (n = 3, mean ± SEM). The increase in particle size was accompanied by a decreased in the amount of cellular uptake of siRNA (Fig. 5b). In particular, the percentage of cells internalizing siRNA was significantly reduced as the result of the increase in the particle size of RNAiMAX (Fig. 5c). These results suggest that the low efficiency of cellular uptake of RNAiMAX was due to the aggregation of particles.

Therefore, we conclude that the small size and high non-aggregability, namely a high stability in the medium, of the YSK12-MEND are advantageous in terms of enhancing cellular uptake by immune cell lines.

## Discussion

Non-viral vectors, especially lipid-based reagents, are widely used for transfecting siRNA into cell lines, because they are easily handled. However, success in human immune cells, also including cell lines, has been limited. In fact, the RNAiMAX was taken up by mouse immune cells (dendritic cells) more efficiently that the YSK12-MEND in a previous report^15^, while, in the case of human immune cells, the RNAiMAX failed to become internalized by the cells (Fig. 3). These findings indicate that the transfection of siRNA to human immune cells is difficult compared to that for mouse immune cells. Thus, gene silencing in human immune cells and cell lines has relied on lentivirus vectors or electroporation methodology^16^,^17^,^18^,^19^,^20^,^21^. There are some reports showing siRNA transfection into human immune cells via the use of non-viral reagents. The following findings have been reported for certain cell types, non-viral reagent, siRNA dose and gene silencing efficiency: 1) Jurkat: protein-based reagent, 50 nM, less than 10%^22^; Lipofectamine 2000 (Invitrogen), 50 nM, no effect^22^; cell-penetrating peptides (CPPs), 50 nM, 75%^23^; RNAiMAX, 100 nM, 25%^23^; Transfuctin (IDT Techonogies), 100 nM, 55%^23^; CPPs, 80 nM, 50%^24^; Hifect (Lonza), 80 nM, no effect^24^; Fugene HD (Promega), 80 nM, no effect^24^. Although the efficient gene silencing in Jurkat cells appeared to be difficult, the gene silencing efficiencies at siRNA doses of 1, 3 and 10 nM by the YSK12-MEND were 75%, 92% and 96%, respectively (Fig. 1a). In this study, the gene silencing efficiency by RNAiMAX was also low (about 20%) and was consistent with other reports (Fig. 1a). 2) THP-1: HiPerFect (Qiagen), 60 nM, 80%^13^; Oligofectamine (Invitrogen), 100 nM, 70%^25^; Dlin-KC2-DMA containing lipid nanoparticle, 250 nM, 80%^12^. The gene silencing efficiencies at siRNA doses of 1, 3 and 10 nM by YSK12-MEND were 57%, 77% and 96%, respectively (Fig. 1b). 3) KG-1: Dlin-KC2-DMA containing lipid nanoparticle (LNP), 250 nM, 30%^12^. Although the Dlin-KC2-DMA containing LNP is a powerful siRNA delivery system, the gene silencing efficiency was low. In our study, RNAiMAX induced only a 12% gene silencing at a siRNA dose of 10 nM (no cytotoxic dose) (Fig. 1c). On the other hand, the gene silencing efficiencies at siRNA doses of 3 and 10 nM (no cytotoxic doses) by the YSK12-MEND were 70% and 87%, respectively (Fig. 1c). 4) NK92: To our knowledge, the use of lipid-based or CPP-based reagents for siRNA transfection has not been reported previous to this study. Nucleofector (Lonza), a type of electroporation method, has been used^26^,^27^. This fact indicates that siRNA transfection to NK92 cells by lipid-based reagents is a substantial challenge. In this study, RNAiMAX also failed to induce significant gene silencing in NK92 cells (Fig. 1d). In contrast, the YSK12-MEND induced a 55% gene silencing effect at a siRNA dose of 10 nM (no cytotoxic dose) (Fig. 1d). This efficiency was lower than those in Jurkat, THP-1 and KG-1 cells. To increase the gene silencing effect in NK92 cells, some modifications of the YSK12-MEND will be needed. Collectively, the YSK12-MEND can be assumed to be a useful siRNA delivery system inducing highly effective gene silencing at low doses in human immune cells, compared with the conventional non-viral vectors.

Cytotoxicity is often a problem in the case of nanoparticles containing a cationic lipid. A typical cationic lipid consists of a cationic head, a linker region and a hydrocarbon backbone. The direct interaction between the cationic head and cell components, especially the cellular membrane, can resulted in cytotoxicity such as cell lysis and necrotic death^28^. In this study, significant cytotoxicities by the YSK12-MEND and RNAiMAX were observed in KG-1 and NK92 cells (Fig. 2c and d). These cytotoxicities appear to be due to the loss of integrity of the plasma membrane by interacting with the cationic head, because the cytotoxicities were independent of the amount of cellular uptake (Fig. 3). In the case of NK92 cells, the gene silencing effect of YSK12-MEND remained at 55% at the siRNA dose that is not cytotoxic. Some modification of the YSK12-MEND will also be needed to address the issue of cytotoxicity. In the case of KG-1 cells, the YSK12-MEND induced sufficient gene silencing at no cytotoxic dose (10 nM) (Fig. 2c). Furthermore, the YSK12-MEND showed no cytotoxicity against Jurkat and THP-1 cells in the experimental dose range (Fig. 2a and b). This suggests that the YSK12-MEND may induce sufficient gene silencing without cytotoxicity in human immune cells.

It is well known that, in the case of suspension cells which consist of several types of immune cells (including cell lines), it is difficult to introduce siRNA using conventional lipid-based or polymer-based delivery systems, but the exact mechanism responsible for this has remained unclear. The results reported herein suggest one possible mechanism, namely particle size and the non-aggregability of particles in the transfection medium. The particle size of the YSK12-MEND in transfection medium was not changed (Fig. 5a). On the other hand, the initial particle size of RNAiMAX after preparation was higher than that of YSK12-MEND, and this particle size increased even more in the transfection medium (Fig. 5a). In addition, the increased particle size impaired cellular uptake (Fig. 5b and c). The aggregation of particles was suspected to induce an increase in particle weight and density, resulting in the inhibition of access to the cells. On the other hand, electrostatic interaction of RNAiMAX with cells appears to be stronger that of YSK12-MEND, because the zeta-potential of RNAiMAX may be much higher than that of YSK12-MEND. Given the low cellular uptake of RNAiMAX, the effect of aggregation appears to exceed the strength of the electrostatic interactions. The findings suggest that the low gene silencing efficiency of conventional lipid-based or polymer-based delivery systems may be due to their low accessibility caused by aggregation. In the case of the YSK12-MEND, it appeared to float in the transfection medium similar to cells, resulting in easy access to cells. The interaction of the YSK12-MEND with cells appeared to be due to the weak cationic charge of the YSK12-MEND. Thus, the small particle size and high non-aggregability in the transfection medium are considered to be important characteristics that are required for the efficient siRNA delivery to immune cells. This insight would be useful for developing delivery systems for human immune cells, while further research will be needed to clarify the detail mechanism responsible for the efficient siRNA delivery by the YSK12-MEND because RNAiMAX is a lipoplex type, making it different from the YSK12-MEND.

The results of the present study show that the YSK12-MEND achieved the effective gene silencing in human immune cell lines, Jurkat, THP-1, KG-1 and NK92 cells. Jurkat and THP-1 cells are widely used in many types of studies. The YSK12-MEND can be a helpful technology for gene functional analysis. NK92 cells have been used for NK-based cancer immune therapy in clinical trials^29^. Although some problems remain, the YSK12-MEND may be applicable for use in NK-based cancer immune therapy in the future. In addition, the YSK12-MEND is expected to induce effective gene silencing in human primary immune cells. Taken together, the YSK12-MEND will serve as a valuable tool for future biological research, and provide a strong platform for the development of siRNA delivery systems to human immune cells.

## Methods

### Materials

YSK12-C4 (6Z, 9Z, 28Z, 31Z)-19-(4-(dimethylamino)butyl) heptatriaconta-6,9,28,31-tetraen-19-ol was synthesized as previously described^15^. Cholesterol were purchased from Avanti Polar Lipids Inc. (Alabaster, AL). 1,2-dimyristoyl-sn-glycerol methoxyethyleneglycol 2000 ether (PEG-DMG) was purchased from NOF Corporation (Tokyo, Japan). RNAiMAX was also obtained from Thermo Fisher Scientific (Waltham, MA). Anti-human GAPDH siRNA (Silencer^®^ GAPDH siRNA) and control siRNA (Silencer^®^ negative control siRNA) were purchased from Thermo Fisher Scientific. Cy5-labled siRNA (Cy5-siRNA, sense: 5′-AcA uGA AGc AGc ACG ACu UT*T-3′; antisense: 5′-AAG UCG UGC UGC UUC AUG UTT Cy5-3′, 2′-OMe are denoted in lower case letters and phoshorothioate linkages are represented by asterisks) was synthesized by BIONEER (Daejeon, Korea)

### Cell lines

Jurkat, THP-1, KG-1 and NK92 cells were purchased from the American Type Culture Collection (Manassas, VA). Jurkat cells were cultured in RPMI 1640 medium containing 10 mM HEPES, 1 mM sodium pyruvate, 100 units/mL penicillin-streptomycin and 10% fetal bovine serum (FBS). THP-1 cells were cultured in RPMI 1640 medium containing 0.05 mM 2- mercaptoethanol, 10 mM HEPES, 1 mM sodium pyruvate, 100 units/mL penicillin-streptomycin and 10% FBS. KG-1 cells were cultured in Iscove’s Modified Dulbecco’s Medium containing 20% FBS. NK92 cells were cultured in α-MEM containing 0.2 mM inositol, 0.1 mM 2- mercaptoethanol, 0.02 mM folic acid, 200 U/ml interleukin 2, 12.5% horse serum and 12.5% FBS.

### Preparation of YSK12-MEND

YSK12-MEND was prepared as previously reported^15^. The YSK12-MEND was composed of YSK12-C4, cholesterol and PEG-DMG (85/15/1 molar ratio). Briefly, 425 nmol of YSK12-C4, 75 nmol of cholesterol and 5 nmol of PEG-DMG were dissolved in 400 μl of 90% (v/v) t-BuOH. 200 μl of a 600 pmol siRNA (siGAPDH or siCtl) solution was gradually added to the lipid solution with vortexing and the mixture was quickly diluted with 2 ml of 20 mM citrate buffer (pH 6.0) to a final concentration of <20% t-BuOH. The residual t-BuOH was replaced with PBS (pH 7.4), resulting in concentrating the YSK12-MEND. The diameter of the MENDs was determined by dynamic light scattering, and zeta potentials were determined by laser-Doppler velocimetry with a ZETASIZER Nano (ZEN3600, Malvern Instruments Ltd., Malvern, WR, UK). The diameter and zeta potential of the YSK12-MENDs were measured in 10 mM HEPES buffer (pH 7.4). The diameter, PDI and zeta-potential of the YSK12-MEND were 150 ± 3 nm, 0.059 ± 0.011 and 8.2 ± 0.4 mV, respectively. The siRNA encapsulation efficiency of YSK12-MEND was determined by a RiboGreen assay as previously described^15^.

### Evaluation of gene silencing activity against GAPDH

The evaluation of gene silencing of GAPDH was performed as reported previously^15^. Jurkat, THP-1, KG-1 or NK92 cells (6.0 × 10^5^ cells) were seeded to 12 well plate and each carrier was added to the cells at siRNA doses of 1–30 nM. The RNAiMAX preparation was carried out following the manufacturer’s instructions. The RNAiMAX reagent (0.3 μl) was mixed with 1 nmol of siRNA. The cells were then incubated for 2 h at 37 °C in 0.5 ml of serum-free OPTI-MEM I. After a 2 h period of incubation, 0.5 ml of culture medium was added to the cells, followed by a further incubation for 22 h. After the incubation, the cells were collected and used for mRNA isolation using a RNeasy Mini Kit (QIAGEN, Hilden, Germany) according the manufacturer’s instructions. Briefly, the DNA contamination in the total RNA was eliminated by a DNase I treatment. The total RNA was then reverse-transcribed using a PrimeScript reverse transcription (RT) reagent Kit (Takara Bio Inc., Shiga, Japan) with oligo-dT primer. Quantitative polymerase chain reaction (PCR) was performed with a Mx3000 P QPCR System (Agilent Technologies, Santa Clara, CA) in 25 μl aliquots of reaction mixures containing cDNA, appropriate pairs of primers and THUNDERBIRD SYBR qPCR Mix (TOYOBO Co., Osaka, Japan). GAPDH level was calculated by the comparative *C*_T_ method using beta actin as endogenous housekeeping genes. The following primer pairs were used: GAPDH: 5′-CCTCTGACTTCAACAGCGAC-3′ (forward); 5′-CGTTGTCATACCAGGAAATGAG-3′ (reverse); beta actin: 5′-CACTCTTCCAGCCTTCCTTC-3′ (forward); 5′-TACAGGTCTTTGCGCATGTC-3′ (reverse).

### Analysis of cytotoxicity

The analysis of cytotoxicity was performed by Premix WST-1 Cell proliferation Assay System (Takara Bio Inc.). Jurkat, THP-1, KG-1 or NK92 cells (9 × 10^4^ cells) were seeded to 96 well plate and each carrier was added to the cells at siRNA doses of 1–100 nM. The cells were then incubated for 2 h at 37 °C in 75 μl of serum-free OPTI-MEM I. After a 2 h period of incubation, 75 μl of culture medium was added to the cells, followed by a further incubation for 22 h. 15 μl of WST-1 assay reagent was then added to each well and the cells were incubated for 1–3 h (Jurkat and THP-1: 1 h; NK92: 2 h; KG-1: 3 h) at 37 °C. After the incubation, the absorbance at 450 nm (reference 630 nm) was measured and used to calculate the relative absorbance (the absorbance of non-treatment was set to 1.0).

### Evaluation of cellular uptake

Jurkat, THP-1, KG-1 or NK92 cells (6.0 × 10^5^ cells) were incubated with the Cy5-siRNA loaded YSK12-MEND or RNAiMAX at siRNA concentrations of 0.3–30 nM for 2 h at 37 °C in 0.5 mL of serum-free OPTI-MEM I in 12 well plate. When we prepared Cy5-siRNA loaded YSK12-MEND or RNAiMAX, a GAPDH siRNA solution containing 10% Cy5-siRNA was used. After the incubation, the cells were collected and washed with PBS containing 20 U/ml heparin. The cells were suspended with FACS buffer (PBS containing 0.1% NaN_3_ and 0.5% bovine serum albumin) and were analyzed by flow cytometer (Gallios, Beckman Coulter, Indianapolis, IN). The data analysis was performed by Kaluza software (Beckman Coulter).

### Drawing of scatter plot between gene silencing efficiency and amount of siRNA in cells

The only relationship between YSK12-MEND and RNAiMAX was shown in the scatter plot. The cell type was not considered. Based on the average values of Fig. 1 (9 values of each carrier), the gene silencing efficiencies (%) were calculated as the ratio of NT value (=100%). The values of FI (GeoMean) at the correspondent siRNA doses in Fig. 2b (9 values of each carrier) were used as the amount of siRNA in cells. The values for FI (GeoMean) were normalized by the value of NT and are shown as relative FI (GeoMean).

### Evaluation of change of particle size

siGAPDH-loaded YSK12-MEND and siGAPDH-loaded RNAiMAX were incubated at a siRNA concentration of 30 nM in OPTI-MEM I at 37 °C, 5% CO_2_, which was the same condition as siRNA transfection experiment. The RNAiMAX preparation was carried out following the manufacturer’s instructions. After 0.5, 1, 2, and 6 h incubation, the diameters were measured with a ZETASIZER Nano.

### Effect of particle aggregation on cellular uptake

Cy5-siRNA loaded RNAiMAX were incubated in OPTI-MEM I at 37 °C, 5% CO_2_ for 1 h or 3 h. These RNAiMAX solutions were added to Jurkat cells (6.0 × 10^5^ cells) at a siRNA concentration of 10 nM and the mixtures were incubated for 37 °C in 0.5 mL of serum-free OPTI-MEM I in 12 well plates. After the incubation, the cells were collected and were washed with PBS containing 20 U/ml heparin. The cells were suspended with FACS buffer and were analyzed by flow cytometer (Gallios). The data analysis was performed by means of the Kaluza software program.

### Statistical Analysis

Comparisons between the two treatments were performed by an unpaired t-test. Statistical analysis of multiple comparisons were performed by one-way ANOVA, followed by the Tukey-Kramer test or Dunnett test. A *P* value of < 0.05 was considered to be a significant difference.

## Additional Information

**How to cite this article**: Nakamura, T. *et al*. Small-sized, stable lipid nanoparticle for the efficient delivery of siRNA to human immune cell lines. *Sci. Rep.* **6**, 37849; doi: 10.1038/srep37849 (2016).

**Publisher's note:** Springer Nature remains neutral with regard to jurisdictional claims in published maps and institutional affiliations.

## Supplementary Material

Supplementary Information
