# Supplementary material for: Small-sized, stable lipid nanoparticle for the efficient delivery of siRNA to human immune cell lines
Source: Sci Rep. 2016 Nov 28;6:37849. doi: 10.1038/srep37849 (PMC5124971; doi:10.1038/srep37849)
Supplement: Supplementary Information [file srep37849-s1.pdf]

Supplementary Information

Title

Small-sized, stable lipid nanoparticles for the efficient delivery of siRNA to human immune cell lines

Author

Takashi Nakamura, Moeka Kuroi, Yuki Fujiwara, Shota Warashina, Yusuke Sato, and Hideyoshi Harashima*

Faculty of Pharmaceutical Sciences, Hokkaido University, Kita-12, Nishi-6, Kita-ku, Sapporo 060-0812, Japan

*Correspondence:

Hideyoshi Harashima, Faculty of Pharmaceutical Sciences, Hokkaido University, Sapporo, Hokkaido 060-0812, Japan.

Telephone: +81-11-706-3919, Fax: +81-11-706-3734

E-mail: harasima@pharm.hokudai.ac.jp

Methods

Western blotting analysis (Figure S1)

Jurkat, THP-1, KG-1 or NK92 cells (6.0×10^5 cells) were seeded on 12 well plates and each carrier was added to the cells at siRNA doses of 1-30 nM. The RNAiMAX was prepared following the manufacturer's instructions. The RNAiMAX reagent (0.3 µl) was mixed with 1 nmol of siRNA. The cells were then incubated for 2 h at 37°C in 0.5 ml of serum-free OPTI-MEM I. After a 2 h incubation period, 0.5 ml of culture medium was added to the cells, followed by a further incubation for 46 h. After the incubation, the cells were collected and washed with PBS. The cells were lysed with 50 µl of RIPA buffer (50 mM Tris-HCl (pH7.4), 1% NP-40, 0.5% Na-deoxyxholate, 0.1% SDS, 150 mM NaCl, 2 mM EDTA) containing 1 µl of protease inhibitor cocktail (Nacalai tesque, Kyoto, Japan) for 30 min on ice. The lysates were then centrifuged and the supernatants were obtained as cell lysates. The cell lysates were heated (95°C, 5 min) with an equal volume of loading buffer (100 mM Tris-HCl (pH 6.8), 4% SDS, 12% 2-mercaptoethanol, 20% glycerol, 0.05% bromophenol blue), and then subjected to 10% SDS-PAGE. After electrophoresis, the proteins were electroblotted onto a Polyvinylidene Fluoride membrane (GVS, Bologna, Italy) and the membranes were then blocked with 2% skim milk. After blocking, a primary antibody from mouse against GAPDH1 (Abcam, Cambridge, UK) and from rabbit against alpha-tubulin (Cell Signaling Technology, Danvers, MA) were used at dilutions of 1:1250 and 1:1000, respectively. Secondary HRP-conjugated anti-mouse (GE Healthcare UK Ltd, Buckinghamshire, England) or anti-rabbit antibodies (Thermo Fisher Scientific, Waltham, MA) were then used at dilutions of 1:1000 and 1:4000, respectively. Blots were developed with Amersham ECL Plus Western Blotting Detection System (GE Healthcare). Immunoreactive bands were visualized using LAS 4000 (Fujifilm, Tokyo, Japan).

Evaluation of cellular uptake (Figure S2)

Jurkat, THP-1, KG-1 or NK92 cells (6.0×10^5 cells) were incubated with Cy5-siRNA loaded YSK12-MEND or RNAiMAX at siRNA concentrations of 0.3-30 nM for 2 h at 37°C in 0.5 mL of serum-free OPTI-MEM I in 12 well plate. When we prepared Cy5-siRNA loaded YSK12-MEND or RNAiMAX, a GAPDH siRNA solution containing 10% Cy5-siRNA was used. After the incubation, the cells were collected and were washed with PBS containing 20 U/ml heparin. The cells were suspended with FACS buffer (PBS containing 0.1% NaN_3 and

0.5% bovine serum albumin) and were analyzed by flow cytometer (Gallios, Beckman Coulter, Indianapolis, IN). The data analysis was performed by Kaluza software (Beckman Coulter).

Evaluation of change of particle size (Figure S3)

siGAPDH-loaded YSK12-MEND and siGAPDH-loaded RNAiMAX were incubated at a siRNA concentration of 30 nM in 10 mM HEPES buffer (pH 7.4) at 37°C, 5% CO_2. The RNAiMAX preparation was carried out following the manufacturer's instructions. After 0.5, 1, 2, and 6 h of incubation, the diameters were measured with a ZETASIZER Nano (ZEN3600, Malvern Instruments Ltd., Malvern, WR, UK).

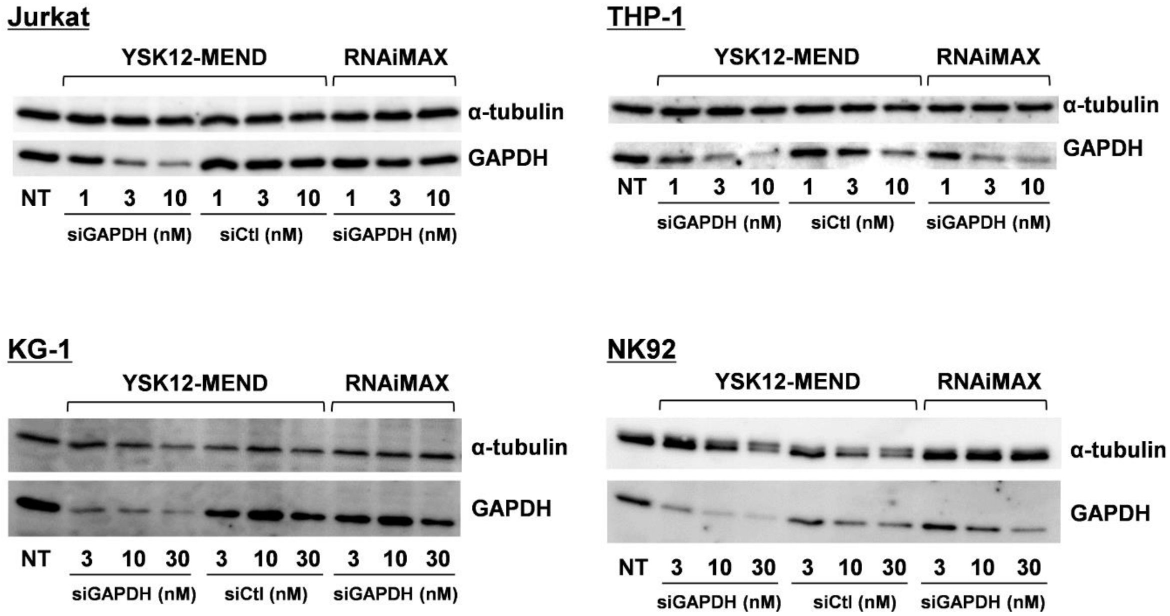

Figure S1. Evaluation of the gene silencing effect by YSK12-MEND at the protein level. Cells were treated with siGAPDH-loaded YSK12-MEND, siCtl-loaded YSK12-MEND or siGAPDH-loaded RNAiMAX. After 48 h, the cells were collected and were analyzed by western blotting. Alpha-tubulin was used as an internal control.

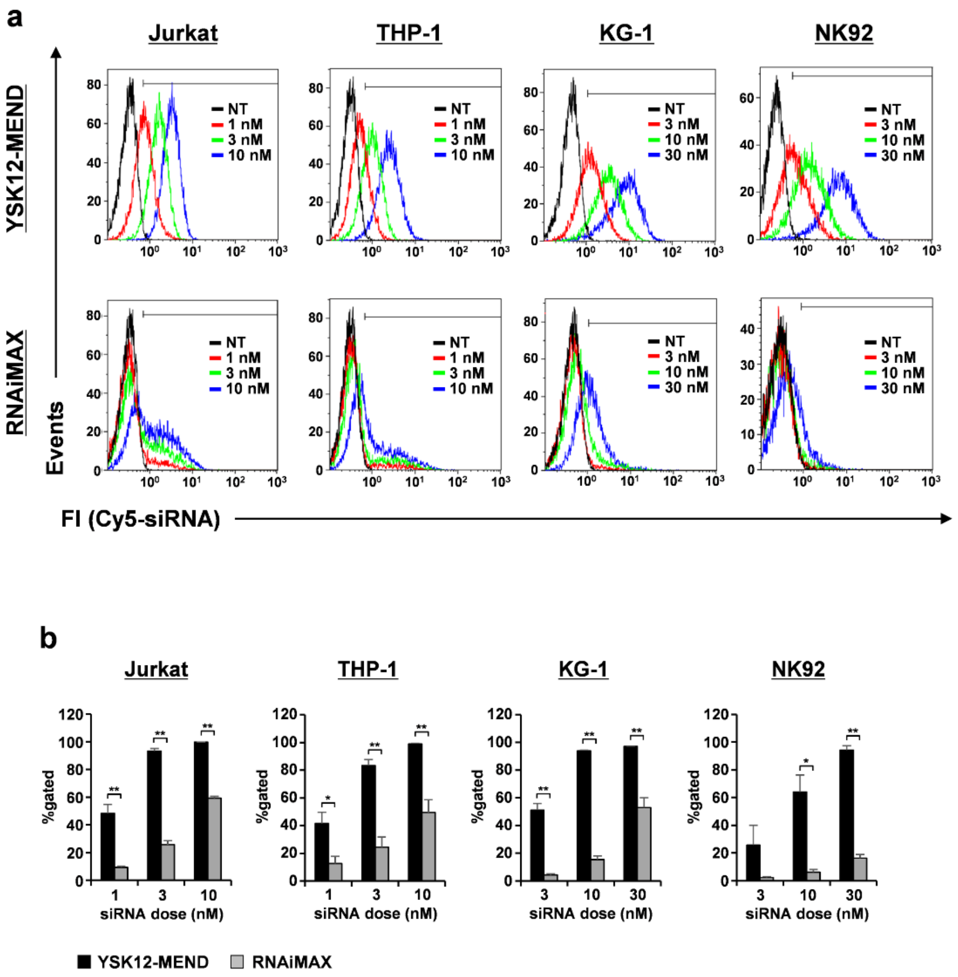

Figure S2. Percentage of human immune cell lines internalizing YSK12-MEND.

Cells were transfected with the YSK12-MEND (Cy5-siRNA) or RNAiMAX (Cy5-siRNA) at siRNA doses of 1-30 nM. After 2 h, the cells were collected and analyzed by flow cytometry. (a) Typical histograms. The single linear region shows the cell population internalizing Cy5-labled siRNA. (b) Average data of cell percentage in the single linear region. Data are mean+SEM (n=3, **P<0.01, *P<0.05).

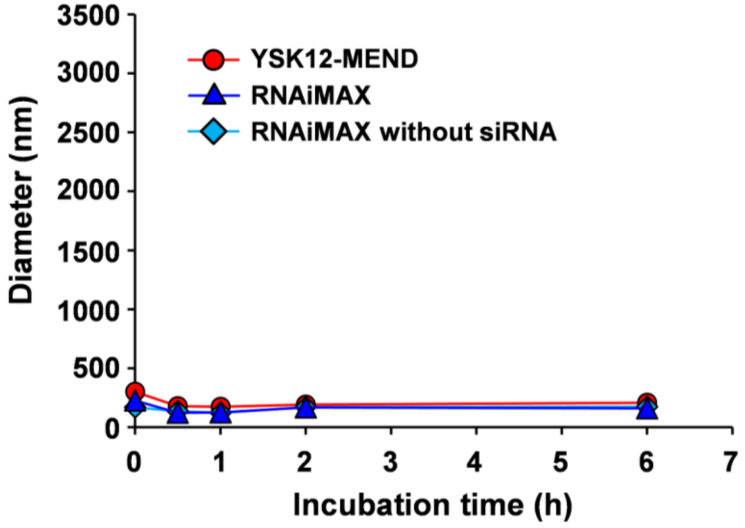

Figure S3. Diameter changes of YSK12-MEND and RNAiMAX in HEPES buffer (pH 7.4).

YSK12-MEND and RNAiMAX were incubated in HEPES buffer (pH 7.4) and the change in diameter was measured. Data are the mean+SEM (n=3).
